# Parapharyngeal metastasis from papillary thyroid microcarcinoma

**DOI:** 10.11604/pamj.2020.37.18.22933

**Published:** 2020-09-04

**Authors:** Khaled Harrathi, Malika El Omri, Rim Fradi, Amel El Korbi, Naourez Kolsi, Rachida Bouatay, Jamel Koubaa

**Affiliations:** 1 Ear, Nose and Throat Department and Cervical Surgery Fattouma Bourguiba Hospital, Medicine University, Monastir, Tunisia

**Keywords:** Metastasis, unknown primary, papillary carcinoma, parapharyngeal metastasis, neck dissection

## Abstract

Carcinoma of the thyroid gland is one of the most commonly encountered endocrine malignancies. Papillary carcinoma is the most common histological type and its pattern of metastasis are usually lymphatic. Lymphatic metastasis to parapharyngeal space is rare and have been reported, so we report the case of a 50-year-old male patient who had an occult papillary carcinoma of the thyroid presented as right lateral node of the neck and a nodal involvement of the right parapharyngeal space.

## Introduction

Thyroid cancer is very commonly encountered in clinical practice [1]. In general, papillary carcinomais the most often encountred histological type and its pattern of metastasis is usually lymphatic [2]. Lymph node dissemination is often reported to the central compartment of the neck followed by the lateral region. Metastasis to the parapharyngeal space seem to be straight forward due to the close anatomical localisation, but a few cases of parapharyngeal involvement have appeared in the literature [3-5]. We present papillary thyroid carcinoma metastasis involving the parapharyngeal space.

## Patient and observation

**Figure 1:**
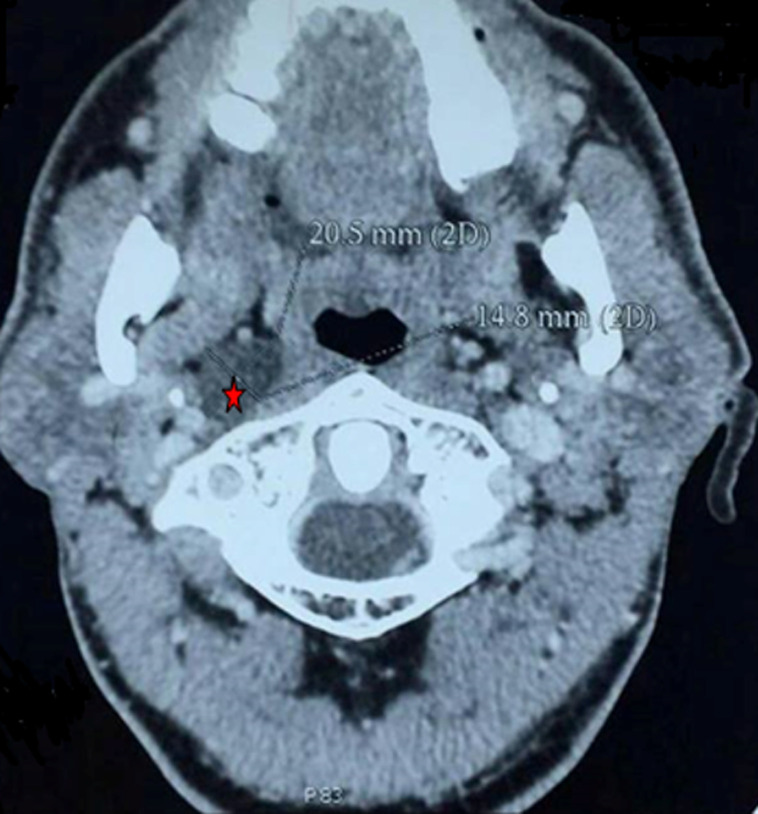
axial post-contrast CT image showing a non-enhanced cystic mass in the right retropharyngeal space

**Figure 2:**
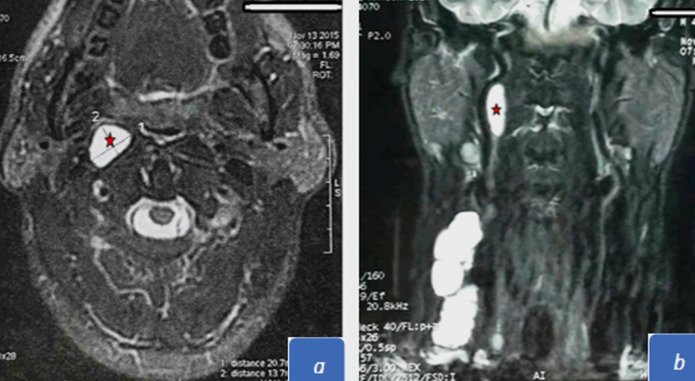
MRI showing enhanced lymph node swelling in the right retropharyngeal space which doesn´t dislocate the intenal carotid artery; a) axial T2 weighted with fat-suppressed; b) coronal T2 weighted with fat-suppressed

A 50-year-old man was referred to our department with a four months history of an enlarging right neck mass. There was no evidence of thyroid dysfunction or any other relevant medical history. He had no associated symptoms of dysphonia, dysphagia or epistaxis. The physical examination revealed a 2*2 cm right high jugular node and a 3*2 cm mid jugular node which were firm and mobile. A detailed examination of the head and neck region showed no intra-oral lesions. The thyroid gland was not palpable and the patient had no other cervical lymphadenopathy. The rhinopharynx exam and the indirect laryngoscopy were normal. The basic investigations including a complete blood count and thyroid function tests were all within the normal range. The cervical ultra sound scan showed two lymph nodes, which were jugulodigastric and mid jugular nodes. These lymph nodes were measuring successively, 45 and 38 mm and contained intra nodal necrosis and microcalcifications. This exam did not find any abnormality of the thyroid gland. The fine needle aspiration cytology (FNAC) of the mass was inconclusive. Owing to clinical suspicion of unknown primary, cervical computed tomography (CT) scan and magnetic resonance imaging (MRI) were requested. In addition to the right cervical nodes identified by the ultrasound, CT scan and MRI demonstrated a 20*15 mm right retropharyngeal node. The CT scan showed a right retropharyngeal mass which was hypodense and cystic. (Figure 1) and the MRI revealed a hyperintense signal on both T2 and T1 sequences that was associated to a moderate homogeneous enhancement (Figure 2). A second FNAC of the right high jugular node was consistent with metastasis from papillary thyroid carcinoma.

**Figure 3:**
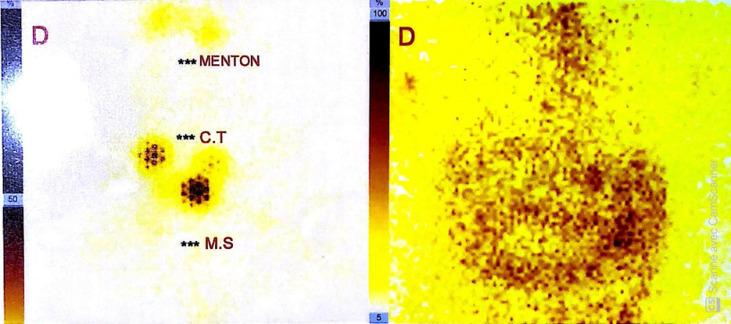
a post-treatment 131I whole body scan showed no uptake by any part of the body

The patient had a total thyroidectomy with a selective neck dissection (Level II, III, IV and VI lymph nodes) and an excision of the parapharyngeal mass through a transoral approach. The incision was made along the anterior tonsillar pillar in the mucosa overlying the lymph node. Right tonsillectomy is performed to facilitate the access. The superior and middle constrictor muscles were identified to enter to the submuscular plane which contain the fibrofatty tissue of the retropharyngeal space. A careful dissection exposed the node within the retropharyngeal space. Finally, the node was mobilized in a pericapsular plane. The postoperative morbidity was minimal and without definitive cranial nerve paralysis. In addition, the histological examination of the thyroidectomy speciman showed a multifocal and encapsulated thyroid microcarcinoma. It found a 0.4 cm nodule in the right lobe and a 0.5 cm nodule in the left lobe of the thyroid. We found also metastatic nodes in the right lateral region of the neck and in the bilateral central compartment. A parapharyngeal metastatic node from papillary thyroid carcinoma was also confirmed. A chest and abdominal CT scan and a bone scan were performed and showed no evidence of metastatic lesions in those parts of the body. So, the tumor was staged T1aN1bM0 according to the TNM classification of thyroid cancer. The patient had radioidine ablation as adjuvent treatment for the tumor. Now he is disease free and he had a non detectable thyroglobulin concentration. The 131 iodine (I) whole body scan showed no uptake by any part of the body (Figure 3) and the neck ultrasound was normal. He is taking Levothyroxine supplements and having regular follow up at 3-monthly intervals two years after surgery.

## Discussion

Papillary thyroid carcinoma is known for its indolent nature. It commonly disseminates through lymphatics. The vascular spread is rare, when occured, it is usually to bone, brain, lungs and soft tissue [6]. The lymphatics drainage of the thyroid gland is performed toward three directions. Inferiorly, these vessels communicate with the upper mediastinal lymph nodes through lymph channels that follow the recurrent laryngeal nerves, the pretracheal and paratracheal lymph nodes. The superomedial lymphatic group connects the upper deep jugular lymph nodes to the prelaryngeal lymph nodes. Lateral lymphatics drainage of the thyroid flow into the middle and lower jugular system [7]. Rouviere, described a lymphatic vessel connecting the upper pole of the thyroid gland to the retropharyngeal lymphatic system. An anatomical dehiscence behind the fascia of the superior constrictor muscle allows the retropharyngeal and parapharyngeal spaces to communicate easily with each other [7,8]. Once they reach the retropharyngeal lymphatics, tumor cells can easily extend to the parapharyngeal space through this weak point. These lymphatic channels, may be the only explanation of the transportation of tumor cells to the parapharyngeal space through the retropharyngeal region [1]. This metastasis is very rare, Desuter reported that 0.43% of thyroid carcinoma had parapharyngeal lymph node metastasis [2]. There are two types of classification for parapharyngeal lymph node metastasis that have been established: primary or secondary cases. The primary cases include T1a thyroid carcinoma, whereas secondary cases include recurrences of thyroid carcinoma that were treated previously.

The diagnosis of metastatic thyroid cancer should be mentioned in cases presenting with isolated cervical lymphadenopathy despite a clinically normal thyroid gland, which is the case in our study. However, further investigation is crucial to exclude other primary tumors of the head and neck regions. The investigation include FNAC. The cytological nature of the neck mass and the possibility of metastasis is initially determined by FNAC [1,9,10]. If FNAC is inconclusive despite the higher possibility of the metastasis, a thorough search for other possible primary sites should be carried out. The imaging studies in combination with FNAC can find lymphadenopathies in retro and parapharyngeal spaces, so we opt for performing CT scan, MRI and FNAC in our case. MRI is more advantageous than the CT in clearly describing the position of the internal carotid artery relative to the tumor [6,11]. Radionuclide scan represents another investigation and has a higher sensitivity and specifity than CT, because it is able to find parapharyngeal metastasis in early stage and detect the small ones that are less than 1 cm [12]. In this medical case, the recommended treatment is surgery associated to radio-ablation and the surgery consists in total thyroidectomy and selective neck dissection [3,13].

However, external radiotherapy is preferable to radio-ablation when bulky residual thyroid tissue is left behind after surgery [14]. Approaches for parapharyngeal space surgery include the transoral, transcervical or transcervical-transparotid approach with or without mandibulotomy [13-15]. The transcervical approach is the most commonly preferred, but Shellenger recommended the transoral approach which is technically safe and feasible [9,14]. We performed a transoral approach in our case. Unfortunately, clinical details on either the extent of follow up or on the status of the patient were not provided in the majority of publications [4,5,16]. In our case, we assured five years of follow-up. This outcome gives a clear confirmation that papillary thyroid carcinoma, despite unusual node involvement, is characterized by good disease-free and overall survival. The follow up should include clinical exam, thyroglobuline, 131I whole body scan and an ultrasound of the neck. Our patient had a non-detectable thyroglobulin concentration. The 131I whole body scan showed no uptake by any part of the body and the neck ultra sound was normal [15]. So towards every elevation of thyroglobuline after thyroidectomy and neck dissection, we should expect metastasis in unusual localisation and we should perform a CT scan of the neck and chest [5,16].

## Conclusion

Lymphatic metastasis of papillary thyroid carcinoma to parapharyngeal space have been rarely reported. In our case, parapharyngeal space involvement was presented, which is a very rare condition.
